# Secondary Syphilis With Concomitant Bullous Pemphigoid: A Case Report

**DOI:** 10.1155/crdm/1071116

**Published:** 2026-06-18

**Authors:** Subi Rijal, Deekshanta Sitaula, Madhu Gyawalee, Vikash Paudel, Monique Kafle, Anupa Khadka, Sanjog Thapa Magar, Baruna Paudel, Aditya Jha, Amit Amatya, Bhaskar Kayastha

**Affiliations:** ^1^ Department of Dermatology and Venereology, Patan Academy of Health Sciences, Lalitpur, Nepal, pahs.edu.np

**Keywords:** bullous pemphigoid, bullous syphilis, secondary syphilis, the great mimicker

## Abstract

A 52‐year‐old woman presented with a three‐month history of pruritic scaly palmoplantar eruptions that progressed to tense bullae over trunk and extremities along with genital mucosal involvement. Serology confirmed syphilis (VDRL reactive at 1:16 and TPHA positive), and biopsy with direct immunofluorescence (DIF) established bullous pemphigoid (BP). Treatment with benzathine penicillin led to resolution of palmoplantar and genital lesions, but there was no improvement of bullous lesions. Treatment with oral doxycycline and prednisolone significantly resolved the bullous eruptions. In adults, secondary syphilis can rarely be present as bullous eruptions, which may mimic autoimmune blistering disorders like BP, thereby posing a diagnostic dilemma. This case uniquely demonstrates BP persisting despite successful syphilis treatment (8‐fold VDRL decrease), requiring ongoing immunosuppression. Unlike previously reported cases where bullous lesions resolved with antibiotics alone, this differential response confirms that syphilis can trigger independent, self‐sustaining autoimmune bullous disease.

## 1. Introduction

Syphilis, a sexually transmitted infection caused by the bacterium *Treponema Pallidum*, remains a global public health challenge with its rising incidence in recent years [1]. Syphilis can be present at different stages: primary, secondary, latent, and tertiary [2]. Primary syphilis usually presents as a solitary, painless “chancre” at the site of inoculation, occurring about 10–90 days after exposure [3]. Secondary syphilis arises 2–8 weeks after the disappearance of primary chancre and is characterized by a wide range of symptoms affecting the skin, mucus membranes, lymph nodes, and other organs due to the hematogenous spread of the bacteria [4, 5]. Syphilis can present with a vast array of clinical manifestations and has been called “the great mimicker.” The most common presentation of secondary syphilis is diffuse symmetric papulosquamous eruption [6]. Vesicular and bullous eruptions may occur in congenital syphilis; however, adult‐onset secondary syphilis presenting with bullous pemphigoid (BP)–like lesions has rarely been reported [7]. We report a rare case of a syphilitic patient presented with cutaneous manifestations clinically and histopathologically consistent with BP.

## 2. Case Presentation

**FIGURE 1:**
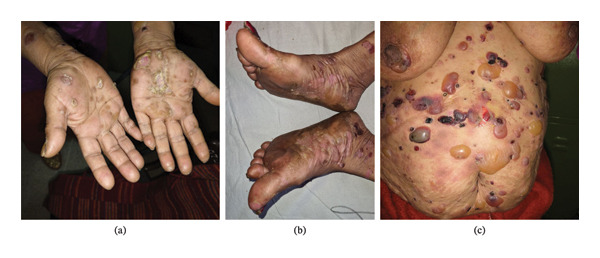
(a) Scaly papules and plaques over bilateral palms. (b) Scaly papules and plaques over soles. (c) Multiple bullae over abdomen, with necrosis.

A 52‐year‐old homemaker presented with a three‐month history of pruritic, scaly eruptions over the palms and soles, accompanied by greyish patches with whitish borders in the genital region. One month prior to presentation, she developed tense bullae over bilateral thighs, which subsequently progressed to involve the trunk and other extremities. She reported her last sexual contact with her husband approximately 6 months before presentation. On specific questioning, she did not recall any preceding painless genital, oral or anal ulcers suggestive of primary syphilis. There was no history of similar lesions in the past and no known history of drug allergies, and she was not on any regular medications. On serological screening, the patient was found to have active syphilis (VDRL reactive at 1:16 and TPHA positive). Subsequently, her husband was also tested and diagnosed with syphilis and was treated accordingly. On examination, there were multiple scaly papules and plaques on the palms and soles, tense bullae—some with necrotic centers—on the trunk and extremities (Figure 1a–c), and greyish patches with whitish borders in the genitalia.

Based on the patient’s history and clinical examination, secondary syphilis with bullous lesions and BP were considered as the primary differential diagnoses. Initial laboratory evaluation revealed a raised total leukocyte count of 19.32 × 10^3^/µL, while routine urine examination was within normal limits. Serological testing confirmed active syphilis, with a reactive VDRL at a titer of 1:16 and a positive TPHA. Screening for other bloodborne infections including HIV, hepatitis B surface antigen (HBsAg), and anti‐hepatitis C virus (anti‐HCV) antibodies were all negative. As the patient could not recall a primary chancre and the exact timing of infection onset remained uncertain, cerebrospinal fluid examination was performed to rule out neurosyphilis. CSF analysis showed nonreactive VDRL and TPHA, excluding neurosyphilis (Table 1).

**TABLE 1:** Summary of key investigations.

Investigation	Result
VDRL	Reactive (1:16)
TPHA	Positive
HIV serology	Negative
Hepatitis B surface antigen (HBsAg)	Negative
Anti‐hepatitis C virus (HCV)	Negative
CSF VDRL/TPHA	Nonreactive
Histopathology	Subepidermal blister with mixed inflammatory infiltrates
Direct immunofluorescence	Linear IgG, C3, and C1 at dermoepidermal junction
Repeat VDRL (3 months)	1:2 (8‐fold decrease)

On admission, serological testing confirmed active syphilis (VDRL reactive at 1:16 and TPHA positive), and the patient was promptly treated with a single intramuscular dose of benzathine penicillin (2.4 MIU). At the same time, skin biopsy and DIF were performed, and further treatment decisions were deferred while awaiting these results. Over the following week, there was a clear improvement in the palmoplantar and genital mucosal lesions; however, the vesiculobullous eruptions over the trunk and extremities persisted, with no appreciable relief in pruritus.

**FIGURE 2:**
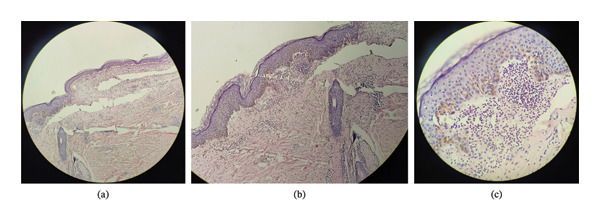
(a) Low‐power photomicrograph showing a subepidermal blister with separation at the dermoepidermal junction and an underlying inflammatory infiltrate within the superficial dermis (hematoxylin and eosin stain, × 100). (b) Section showing subepidermal blister formation with inflammatory infiltrate extending along the dermoepidermal junction (hematoxylin and eosin stain, × 200). (c) Higher magnification view demonstrating a subepidermal cleft with a dense mixed inflammatory infiltrate (hematoxylin and eosin stain, × 400).

After 7 days, the histopathological examination of an intact blister demonstrated subepidermal separation with a mixed inflammatory infiltrate (Figure 2a–c), and DIF revealed linear deposition of IgG, C3, and C1 along the dermoepidermal junction, confirming a diagnosis of BP. In light of the persistent bullous disease and definitive immunopathological findings, systemic therapy was subsequently escalated.

**FIGURE 3:**
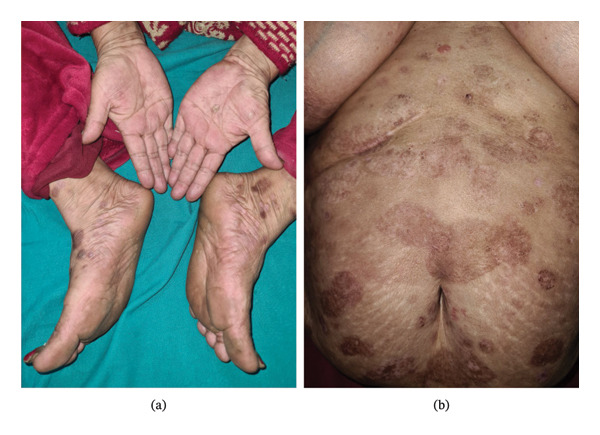
(a) Resolution of lesions of soles after 3^rd^ week of treatment. (b) Resolution of lesions of abdomen after 3 weeks of treatment.

Oral prednisolone was initiated at a dose of 30 mg/day along with oral doxycycline (100 mg twice daily) as adjunctive therapy for BPs, leveraging its anti‐inflammatory properties. Doxycycline was continued for 3 weeks; however, as the bullous lesions showed limited initial response, doxycycline was discontinued, and treatment was continued with corticosteroids along with the steroid‐sparing immunosuppressant azathioprine. While pruritus had shown minimal improvement following penicillin therapy, there was marked symptomatic relief within 1 week of initiating systemic corticosteroids. The vesiculobullous lesions gradually resolved, with no new blister formation observed by the third weekly follow‐up visit. The healed lesions showed postinflammatory hyperpigmentation without evidence of scarring, consistent with typical healing in BPs (Figure 3a,b).

Prednisolone was gradually tapered over the subsequent 4–6 weeks based on clinical response, without evidence of relapse during tapering. Azathioprine was introduced at approximately 2 weeks as a steroid‐sparing agent to facilitate gradual prednisolone taper while maintaining disease control. Given that azathioprine requires 6–8 weeks to achieve full therapeutic effect, early introduction allowed for more effective steroid‐sparing during dose reduction. At the 3‐week follow‐up visit, the patient remained clinically stable, with sustained resolution of active bullae and no recurrence of disease. Repeat VDRL testing at 3 months posttreatment demonstrated an 8‐fold decrease in titer (from 1:16 to 1:2), confirming adequate serological response to syphilis treatment.

## 3. Discussion

Bullous eruptions in secondary syphilis are uncommon in adults and pose significant diagnostic challenges. While vesiculobullous presentations are rare, several case reports have documented bullous or vesiculobullous secondary syphilis in adults, with varying presentations, immunopathological findings, and treatment responses (Table 2).

**TABLE 2:** Comparison of reported cases of bullous/vesiculobullous secondary syphilis in adults.

Ref. no.	Author (year)	Age/sex	Clinical presentation	DIF/serology	Histopathology	Treatment	Outcome
[6]	Kopelman et al. (2019)	18/M	Pemphigus‐like, vesicles and bullae on torso	DIF: Not performed; Serum: Negative for pemphigus antibodies	Acantholysis, neutrophilic and eosinophilic infiltration	Penicillin; no immunosuppression	Complete resolution
[7]	Lawrence and Saxe (1992)	40/M	Bullous eruption, trunk and limbs	DIF: Positive (linear IgG, C3 at basement membrane)	Subepidermal blister, perivascular infiltrate with eosinophils, plasma cells	Procaine penicillin IM × 10 days; no immunosuppression	Rapid permanent resolution
[8]	Schnirring‐Judge et al. (2011)	41/F	Vesiculobullous, plantar feet	DIF: Negative	NR	Penicillin; no immunosuppression	Complete resolution
[9]	Markiewicz et al. (2025)	46/M	Vesiculobullous, polymorphic, recurrent	DIF: Negative for bullous diseases (only C1q deposition)	Subcorneal and subepidermal blisters, lymphocytes, eosinophils, neutrophils	Benzathine penicillin; no immunosuppression	Clinical improvement
[10]	Stone et al. (2023)	60s/M	Pemphigus‐like, diffuse eroded papules/plaques, mucosal involvement	DIF: Positive (focal granular IgG on epithelial cells)	Acantholysis, eosinophilic infiltrate	IV penicillin G 24 million units/day × 14 days; no immunosuppression	Complete resolution
[11]	Kazlouskaya et al. (2014)	3 cases	Pustular, generalized	NR	Pustular eruptions	Penicillin; no immunosuppression	Complete resolution in all 3 cases
	Present case (2025)	52/F	Bullous eruptions on trunk/extremities + palmoplantar scaly papules/plaques	DIF: Positive (linear IgG, C3, C1 at dermoepidermal junction)	Subepidermal blister, inflammatory infiltrate with eosinophils	Benzathine penicillin + Prednisolone + Doxycycline + Azathioprine; YES immunosuppression	Syphilis resolved (VDRL 1:16 ⟶ 1:2, 8‐fold decrease); bullous pemphigoid persisted, requiring ongoing immunosuppression

Lawrence and Saxe reported a 40‐year‐old man who presented with a two‐week course of fluid‐filled bullae over the trunk and limbs. Histological and immunofluorescence findings were consistent with BP (linear IgG and C3 deposition at the dermoepidermal junction). Remarkably, despite the BP‐like immunopathology, the lesions resolved completely following a 10‐day course of intramuscular procaine penicillin without any additional immunosuppressive therapy, suggesting that the bullous eruption was directly attributable to the syphilitic infection [7]. Kopelman et al. described an 18‐year‐old man with pemphigus‐like lesions including vesicles and bullae on the torso, accompanied by acantholysis on histopathology. Similarly, these lesions were resolved with antibiotic treatment alone [6]. Schnirring‐Judge et al. described a 41‐year‐old woman with a bullous eruption on the plantar aspect of the foot, initially misdiagnosed as a bacterial infection. This case, identified as secondary syphilis based on positive serology, showed negative immunofluorescence findings and resolved with antibiotic therapy [8]. More recently, Markiewicz et al. reported a 46‐year‐old man with recurrent polymorphic eruptions, predominantly vesiculobullous in nature. DIF was negative for autoimmune blistering disorders (showing only nonspecific C1q deposition). The patient initially experienced worsening of lesions during early treatment but subsequently improved with benzathine penicillin, without requiring immunosuppressive therapy [9]. Remarkably, Stone et al. reported a case with positive DIF showing focal granular IgG deposition on epithelial cells—a pemphigus‐like immunopathological pattern—in a patient with both secondary syphilis and neurosyphilis who nonetheless achieved complete resolution with high‐dose intravenous penicillin without immunosuppressive therapy [10]. Pustular variants have also been reported. Kazlouskaya et al. described three cases of pustular secondary syphilis, highlighting this as an extremely rare manifestation that can be mistaken for other pustular dermatoses, all of which resolved with penicillin therapy [11].

In stark contrast to all previously reported cases, our patient demonstrated a unique pattern: while the palmoplantar papulosquamous lesions and genital lesions resolved following benzathine penicillin treatment (with confirmed serological cure showing 8‐fold decrease in VDRL titer from 1:16 to 1:2), the bullous lesions persisted and required ongoing immunosuppressive therapy with corticosteroids, doxycycline, and azathioprine. This differential treatment response has not been previously reported and strongly suggests that our patient had true BP coexisting with or triggered by syphilis rather than transient bullous eruptions solely attributable to the infection itself. While the syphilitic infection may have initiated or unmasked the autoimmune response, the BP became an independent disease process requiring sustained immunosuppression.

Development of BP has been associated with various infectious and parasitic agents like human herpes virus, HIV, Hepatitis B and C, *Toxoplasma gondii*, and *Sarcoptes scabiei* [12]. However, there is insufficient evidence to establish a direct causal relationship between syphilis and development of BP. The absence of drug‐induced triggers in our patient, along with the temporal association between syphilis infection and BP development, further supports the hypothesis that infectious triggers—particularly syphilis—may play a role in initiating autoimmune blistering disorders in susceptible individuals. The characteristic healing pattern without scarring in our patient also confirms the diagnosis of BP and excludes other subepidermal blistering conditions such as epidermolysis bullosa acquisita. There is a rare bullous variant of congenital syphilis, known as syphilitic pemphigus, occurring mostly on palms and soles, but may be generalized [13]. It may clinically mimic autoimmune BP but is a distinct entity caused by the infection rather than true BP triggered by syphilis. The protean symptoms of secondary syphilis are due to human immune responses against the spirochetes and, more importantly, activation of macrophages by sensitized CD4‐T cells [14].

In BP, the hemidesmosomal proteins BPAG1 (BP230) and BPAG2 (BP180) are the target of autoantibodies, which activate complement, degranulate mast cells, and attract neutrophils and eosinophils. These cells then release proteolytic enzymes that break down the dermal–epidermal junction and cause subepidermal blisters [15]. According to a few published articles on bullous diseases after hepatitis B vaccination, the HBsAg may cause bullous diseases either by molecular mimicry, in which antigenic components of the recombinant HBV vaccine or the hepatitis B virus mimic host antigens and cause the production of cross‐reactive antibodies, or by nonspecific immune reactivation, which unmasks the latent bullous disorders [16, 17].

For syphilis, a similar mechanism can be hypothesized: *Treponema pallidum* antigens and host basement membrane components may molecularly mimic one another, causing cross‐reactive immune responses that could exacerbate or unmask BP in predisposed individuals through nonspecific immune activation or latent disease reactivation.

BP after syphilis infection can be attributed to molecular mimicry, where *Treponema Pallidum* antigens, particularly the 47‐kDa lipoprotein (Tpp47), share structural motifs with mammalian fibronectins and collagens. As BP180 (collagen XVII), a key autoantigen in BP, is also a collagen, there remains the possibility of cross‐reactivity [18]. Observational studies have highlighted an association between reactive syphilis serology and elevated anti‐BP180 antibodies, even in the absence of overt BP symptoms, hinting this subclinical autoimmune trigger in susceptible individuals [19].

BP following secondary syphilis may also result from complex nonspecific immune activation involving both innate and adaptive immune responses. *Treponema pallidum* uses complex immune evasion strategies to stay in the host and evade immune clearance. Among these mechanisms are the antigenic variety of surface proteins (like TprK), the low number of proteins exposed to the surface (like Tp92), and the modification of host immune cell activities (like causing monocytes to undergo apoptosis and extending neutrophil survival). The pathogen’s outer membrane protein Tp92 stimulates endothelial cells and increases inflammation via cytokine production; however, it also inhibits host immunity by limiting immunogenic epitope exposure, resulting in a chronic inflammatory milieu conducive to persistent infection [20]. Chronic immune stimulation induced by syphilis can disrupt immune tolerance and potentially unmask or trigger autoimmune responses like BPs in genetically or immunologically susceptible people [12].

On the other hand, there may not be a direct causal link between the coexistence of BP and secondary syphilis in this instance; rather, it may be purely coincidental. While the treatment response suggests two distinct entities, it remains a possibility that the syphilitic infection initiated an autoimmune cascade that became self‐perpetuating, explaining the need for ongoing immunosuppression. Further studies are recommended to clarify the exact immunopathological pathways by which syphilis may trigger/exacerbate BPs.

This case contributes to the limited literature on bullous secondary syphilis, which is rare and difficult to diagnose. It emphasizes the significance of a thorough clinical, serological, histological, and immunopathological examination in patients with atypical bullous eruptions, particularly those with risk factors for syphilis. While syphilis may cause or intensify such bullous manifestations, the presence of these conditions may be coincidental too. Careful and detailed assessment is thus required to effectively diagnose and manage these complex situations, regardless of the underlying etiology.

## Funding

No funding was obtained for the study.

## Consent

A written informed consent was obtained from the patient.

## Conflicts of Interest

The authors declare no conflicts of interest.

## Data Availability

The data that support the findings of this study are available on request from the corresponding author. The data are not publicly available due to privacy or ethical restrictions.
